# The Vitamin D status is associated with serum C-reactive protein and adhesion molecules in patients with renal cell carcinoma

**DOI:** 10.1038/s41598-019-53395-9

**Published:** 2019-11-13

**Authors:** Shen Xu, Jin Song, Zhi-Hui Zhang, Lin Fu, Lan Gao, Dong-Dong Xie, De-Xin Yu, De-Xiang Xu, Guo-Ping Sun

**Affiliations:** 1 Department of Oncology, First Affiliated Hospital, Anhui Medical University, Hefei, 230022 China; 2 Department of Urology, Second Affiliated Hospital, Anhui Medical University, Hefei, 230601 China; 3 Department of Toxicology, School of Public Health, Anhui Medical University, Hefei, 230032 China; 4 Key Laboratory of Environmental Toxicology of Anhui Higher Education Institutes, Anhui Medical University, Hefei, 230032 China

**Keywords:** Inflammation, Endocrinology

## Abstract

Low vitamin D status is associated with an increased risk of renal cell carcinoma (RCC). This study investigated the association of vitamin D status with serum C-reactive protein (CRP) and adhesion molecules among RCC patients. Fifty newly diagnosed RCC patients and 100 age- and sex-matched controls were recruited. As expected, serum 25(OH)D level was lower in RCC patients than in controls. By contrast, serum levels of CRP, an inflammatory molecule, and ICAM, LAMA4 and EpCAM, three adhesion molecules, were higher in RCC patients than in controls. All RCC patients were divided into two groups: H-VitD (>20 ng/ml) or L-VitD (<20 ng/ml). Interestingly, the levels of serum CRP and all adhesion molecules were higher in RCC patients with L-VitD than those with H-VitD. Nuclear vitamin D receptor (VDR) was downregulated and nuclear factor kappa B (NF-κB) was activated in cancerous tissues. The *in vitro* experiments found that VitD3 suppressed NF-κB activation and adhesion molecules in RCC cells. Moreover, VitD3 suppressed NF-κB through reinforcing physical interaction between VDR and NF-κB p65 subunit in RCC cells. These results provide a mechanistic explanation for the association among low vitamin D status, local inflammation and increased expression of adhesion molecules among RCC patients.

## Introduction

Renal cell carcinoma (RCC) is common malignant tumor in urinary tract, among which clear cell RCC (ccRCC) is the most common subtype^1,2^. Increasing data suggest that hypertension, obesity, long-term sodium intake, high glycemic index diet, and cigarette smoking are the major risk factors for RCC^3–7^. Numerous studies have demonstrated that inflammatory molecules, such as C-reactive protein (CRP), promote RCC progression^8–10^. Other studies have indicated that inflammatory cytokines, such as tumor necrosis factor alpha (TNF-α), lead to a poor prognosis^11^. On the other hand, adhesion molecules, such as intercellular adhesion molecule (ICAM)-1, vascular cell adhesion molecule (VCAM)-1, melanoma cell adhesion molecule (MCAM), and epithelial cell adhesion molecule (EpCAM), are involved in invasion and migration of malignant tumors^12–15^. In addition, MCAM and EpCAM are prognostic molecular markers in patients with malignant tumors^16–24^. Nevertheless, which factors up-regulate inflammatory molecules and adhesion molecules in cancer cells have not yet been elucidated.

Vitamin D is a secosteroid hormone for calcium and phosphorus metabolism^25^. Several recent studies demonstrate that vitamin D regulates cell proliferation and differentiation, immune response. In addition, vitamin D has anti-cancer, anti-inflammatory and anti-oxidative effects^26–30^. Vitamin D deficiency (VDD) is defined as lower than 20 ng/ml of 25(OH)D^31,32^. Numerous epidemiological reports demonstrated that VDD was positively linked with cancer incidence and a poor clinical prognosis^33–36^. Recently, a large epidemiological investigation showed that VDD was associated with an elevated risk of RCC in men and women^37^. Vitamin D3 (VitD3) is metabolized to 25(OH)D3 by cytochrome P450 (CYP)2R1 in the liver and then metabolized into 1,25(OH)2D3 by CYP27B1 in the kidney^38^. The effects of vitamin D3 are mediated by vitamin D receptor (VDR)^39^. Indeed, CYP27B1 and VDR are highly expressed in human kidney^40^. Therefore, it is especially interesting whether there is an association between VDD and increased inflammatory and adhesion molecules among RCC patients.

The purpose of this study was to analyze the link among serum 25(OH)D, local inflammatory signaling and increased expression of adhesion molecules among RCC patients. We found that serum 25(OH)D level were lower in RCC patients than in controls. By contrary, the levels of serum CRP and several adhesion molecules were elevated in RCC patients. The present study suggests a link among low vitamin D status, local inflammation and increased expression of adhesion molecules in RCC patients.

## Materials and Methods

### Study participants

Total 50 newly diagnosed RCC patients, whose ages ranged from 40 to 75 years old, were recruited as cases. All cases were diagnosed as RCC patients for the first time between January 2013 and December 2014. RCC was confirmed by histopathology. Total 100 control subjects were from physical examination at the Second Affiliated Hospital of the Anhui Medical University. Two control subjects were matched with one RCC case regarding age (within 2 years), sex and season of blood sample. Individuals with a history of cystic nephropathy, tuberous sclerosis, and severe kidney disease were excluded from this study. Patients were staged using the AJCC TNM system^41^. Tumors were graded according to the Fuhrman system^42^. All slides were examined by two pathologists to ensure diagnostic correction. Serum collection and research procedure obtained approval from the ethics committee of Anhui Medical University. Oral and written consents were obtained from all subjects. All participants signed informed consent forms and were told they could quit at any time. All methods, including methods mentioned below, followed the relevant guidelines and regulations.

### Reagents

Antibodies against MCAM, LAMA4, EpCAM and VDR were from Cell Signaling Technology (Beverley, MA). Antibodies against NF-κB p65, p-IκB and β-actin were from Santa Cruz Biotechnologies (Santa Cruz, CA). Chemiluminescence detection kit was from Pierce Biotechnology (Rockford, IL). All other reagents were purchased from Sigma Chemical Co. (St. Louis, MO) if not otherwise stated.

### Biochemical measurement

Venous blood was drawn in the morning with more than 8 hours fasting. Serum creatinine, uric acid, urea nitrogen, calcium and serum phosphorus were measured with automatic biochemical analyzer (KHBZY-1200).

### Radioimmunoassay (RIA)

Serum 25(OH)D concentration was measured by RIA using a commercial kit (DiaSorin Inc, Stillwater, MN, USA) as our previous study^43^. Serum 25(OH)D concentrations were expressed as ng/ml. Less than 20 ng/mL for 25(OH)D level was defined as VDD.

### Cell culture and treatments

ACHN cells and 786-o cells are two types of renal cell cancer cell lines that are from the cell bank of the Chinese Academy of Sciences (Shanghai).The cells were inoculated in a 10 cm culture dish and cultured in 5% carbon dioxide at 37 °C with 100U/ ml penicillin-streptomycin and 10% serum MEM/EBSS (HyClone, for ACHN) or RPMI Medium Modified (HyClone, for 786-O) medium. When the cells grew to 80% full, the mediums were replaced by serum-free mediums. 1,25(OH)D2 was pretreated for 24 hours before LPS was added. Supernatant was collected after 6 hours for ELISA. Cells were collected for Western blot and Co-IP analysis.

### Co-immunoprecipitation (Co-IP)

The collected cells were washed twice with PBS and then lysed for 20 min with 400 μl lysis buffer to obtain total protein. The protein was quantified by BCA Protein Quantitation Kit, and 1000 μg Protein was diluted to 1.2 ml. About 20 μl protein A/G-agarose was added for incubation for 2 h, and supernatant was taken. The supernatant was added with 2 μg anti-VDR antibody, and after 3 h incubation, 40 μl protein A/G-agarose was added for incubation. After 10 h, agarose was collected, washed, and boiled for 10 min with 60 μl loading buffer. Supernatant was collected for immunoblots using VDR and NF-κB p65 antibodies.

### Enzyme-linked immunosorbent assay (ELISA) Measurements of CRP and adhesion molecules

CRP and adhesion molecules (ICAM-1, VCAM-1, MCAM, LAMA4 and EpCAM) were measured using ELISA kits (R&D Systems, Abingdon, Oxon, UK) according to the manufacturer’s protocol.

### Immunohistochemistry (IHC)

The methods of IHC referred to our previous study with minor modification^43^. Briefly, human RCC specimens were fixed in 4% formalin and embedded in paraffin according to the standard procedure. For IHC, paraffin-embedded renal sections were deparaffinized and rehydrated in a graded ethanol series. After antigen retrieval and quenching of endogenous peroxidase, sections were incubated with specific antibodies (MCAM and EpCAM) at 4 °C overnight. The color reaction was developed with HRP-linked polymer detection system and counterstaining with hematoxylin. Tissue sample collection and research procedure obtained approval from the ethics committee of Anhui Medical University.

### Western blot

Eight specimens were randomly selected from cancer patients, whose mean level of serum 25(OH)D was 20.73 ng/ml. Tissue lysate and protein extraction referred to the previous study^43^. For Western blot, 20 μg of protein in loading buffer was subjected to electrophoresis in 12.5% SDS-PAGE for 3 h. The gel was transferred electrophoretically onto a PVDF membrane (Immobilon-P; Millipore powdered milk in Dulbecco’s PBS). The membranes were blocked by nonfat milk and then incubated for 2 h using following antibodies: either VDR or p-IκB or p-p65. For total proteins, β-actin was used as a loading control. For nuclear protein, lamin A/C was used as a loading control. After washed in Dulbecco’s PBS-T for four times and PBS once, the membrane was incubated with goat anti-rabbit or horse anti-goat IgG antibody for 2 h. The membrane was then washed 4 times in Dulbecco’s PBS-T for 10 min and PBS for 10 min, followed by signal development using an enhanced chemiluminescence (ECL) detection kit.

### Statistical analysis

The difference between two independent groups was compared using two student *t* test or the Mann-Whitney U-test. Comparative analyses of categorical variables were carried out by the chi-square test. The link between 25(OH)D and adhesion molecules was analyzed using linear correlation. A *P* value of less than 0.05 was considered statistically significant.

## Results

### Demographic characteristics and grades of renal cell carcinoma

The demographic characteristics are shown in Table 1. As expected, no difference on mean age and sex composition was observed between RCC patients and controls. There was no difference on serum creatinine, uric acid and phosphorus levels between two groups. BMI index and serum urea nitrogen were higher in RCC patients than those of controls (Table 1). By contrary, the levels of serum calcium were lower in RCC patients than those of controls (Table 1). The number of cases at different stages and grades was then analyzed. As shown in Table 2, 60% (30/50) of RCC patients belonged to G1/2 and 40% (20/50) belonged to G3/4. Moreover, 68% (34/50) of RCC patients belonged to T1a/b (34/50, 68%). In addition, 18% (9/50) of RCC patients belonged to T2 and only 14% (7/50) belonged to T3/4 (Table 2).

**Table 1:** The demographic and serum biochemical parameters between cases and controls

	Control (n = 100)	Case (n = 50)	P value
Mean age (years), mean (SD)	56.55 (11.57)	56.88 (11.91)	0.871
Sex, male (%)	73 (73)	36 (72)	0.897
BMI (kg/m^2^)	21.18 (2.57)	24.26 (3.24)	<0.001
Serum creatinine (μmol/L)	80.09 (18.12)	85.72 (31.89)	0.706
Serum urea nitrogen (mmol/L)	5.03 (1.00)	5.81 (1.53)	<0.001
Serum uric acid (mmol/L)	375.35 (96.70)	381.11 (97.16)	0.704
Serum calcium (mmol/L)	2.27 (0.13)	2.21 (0.09)	0.039
Serum phosphorus (mmol/L)	1.15 (0.24)	1.18 (0.26)	0.737

**Table 2:** Fuhrman Grades and AJCC TNM Stages

	Case (n = 50)
**Fuhrman Grades (%)**	
Fuhrman I	2 (4)
Fuhrman II	28 (56)
Fuhrman III	13 (26)
Fuhrman IV	7 (14)
**AJCC TNM Stages (%)**	
T1a	12 (24)
T1b	22 (44)
T2	9 (18)
T3/4	7 (14)

### Serum 25(OH)D is reduced in RCC patients

**Figure 1:**
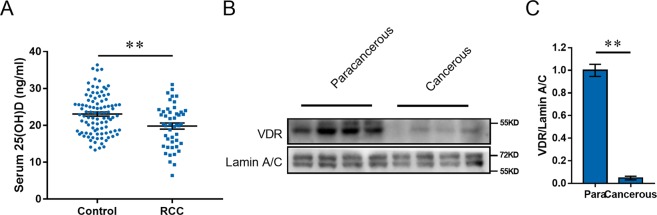
Serum 25(OH)D level and VDR distribution among RCC patients. (**A**) Serum 25(OH)D level in RCC patients and controls. Serum 25(OH)D was measured by RIA. All data were expressed as means ± S.E.M. **P < 0.01. (N = 50 for RCC patients; N = 100 for control subjects). (**B**,**C**) Nuclear VDR level was measured using Western blot. (**B**) A representative gel for VDR (upper panel) and Lamin A/C (lower panel) was shown. (**C**) VDR/Lamin A/C. The grouping of blots cropped from different gels of same samples. All data were expressed as means ± S.E.M. (N = 8). **P < 0.01.

Serum 25(OH)D was analyzed among all subjects. As shown in Fig. 1A, 25(OH)D concentration was 20.73 ng/ml in RCC patients, significantly lower than 23.02 ng/ml in controls. VDD is defined by most experts as a 25(OH)D level of less than 20 ng/ml. A 25(OH)D level of greater than 20 ng/ml and less than 30 ng/ml is defined as vitamin D insufficiency (VDI), and 30 ng/ml or greater is defined as vitamin D sufficiency (VDS)^32,44^. We compared the vitamin D status between RCC patients and controls. We found that the proportion of subjects with VDD (25(OH)D < 20 ng/ml), VDI (20 ≤ 25(OH)D < 30 ng/m) and VDS (25(OH)D ≥ 30 ng/ml) in RCC patients were 44%, 48% and 8%, respectively. The proportion of subjects with VDD, VDI and VDS in controls were 34%, 54% and 12%, respectively. Although there is no significant difference, the proportion of RCC patients with VDD has an increasing trend. Accumulating data have demonstrated that overweight/obesity influences vitamin D status^45,46^. The present study found that BMI index of RCC patients was higher than that of controls. Thus, we further analyzed the association between RCC patients and serum 25 (OH) by multivariable logistic regression, adjusting for BMI. The results are presented in Table 3. As expected, a negative association between serum 25(OH)D and RCC was observed (adjusted OR: 0.946; *P* < 0.05).

**Table 3:** Multivariable logistic regression analysis of correlation between RCC and serum 25(OH)D concentration.

Variable	β	Wald	P	OR (95%C.I.)
Unadjusted	0.053	3.684	0.055	0.948(0.898, 1.001)
Adjusted^a^	0.055	3.905	0.048	0.946(0.896, 0.999)

Note: β: regression coeffcient; Wald: Wald chi-square value; OR: odds ratio.

^a^Adjusted for BMI and age.

### Nuclear VDR is reduced in cancerous tissues among RCC patients

Nuclear VDR levels were analyzed among RCC patients. As expected, nuclear VDR was reduced in cancerous tissue (Fig. 1B,C).

### The levels of serum CRP and adhesion molecules are increased in RCC patients

**Figure 2:**
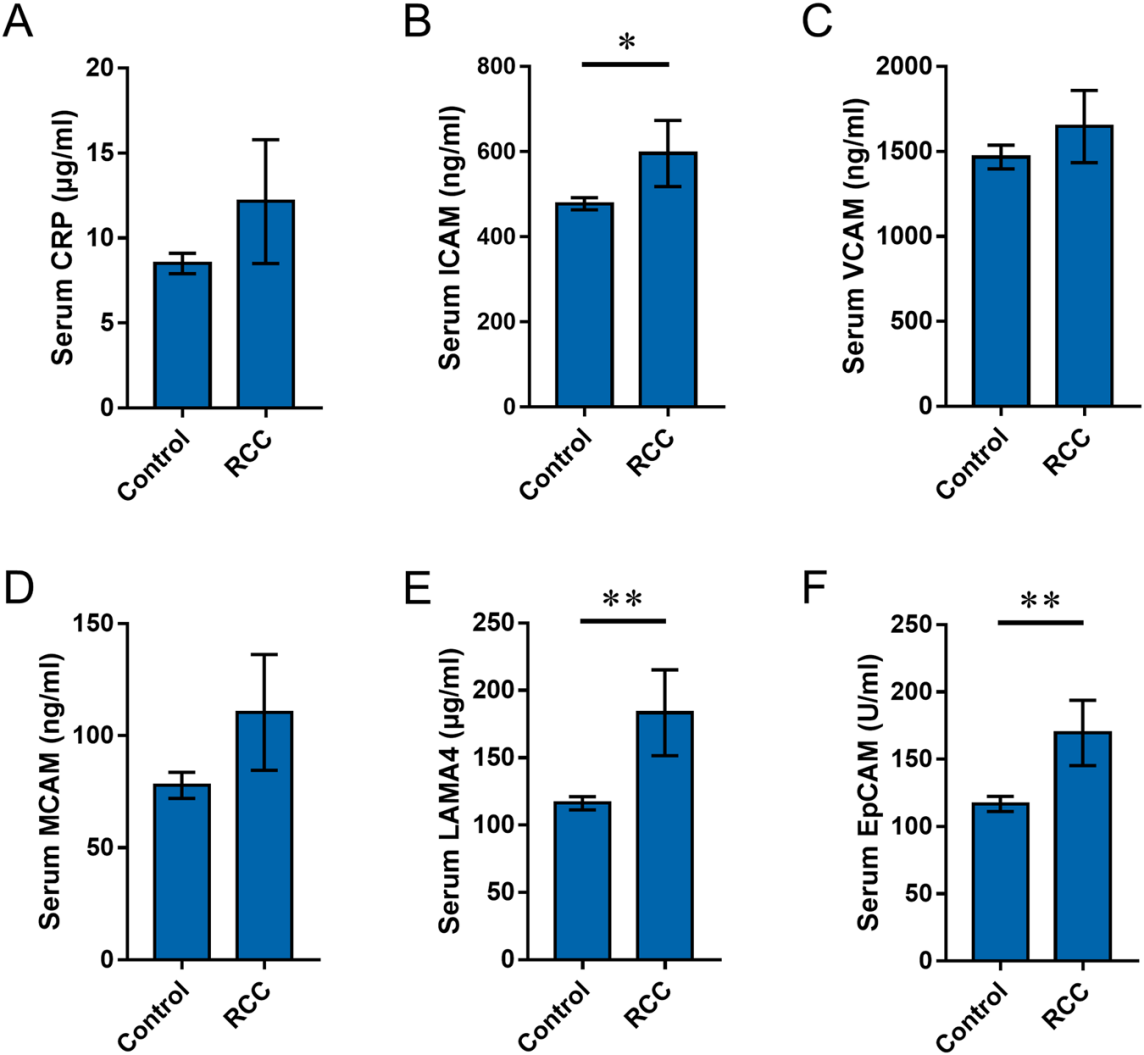
Serum CRP and adhesion molecule levels in RCC patients and controls. Serum CRP and adhesion molecules were measured using ELISA. Serum CRP, ICAM, VCAM, MCAM, LAMA4 and EpCAM levels were compared between RCC patients and controls. (N = 50 for RCC patients; N = 100 for control subjects). (A) CRP; (**B**) ICAM; (**C**) VCAM; (**D**) MCAM; (**E**) LAMA4; (**F**) EpCAM. All data were expressed as means ± S.E.M. *P < 0.05, **P < 0.01.

**Figure 3:**
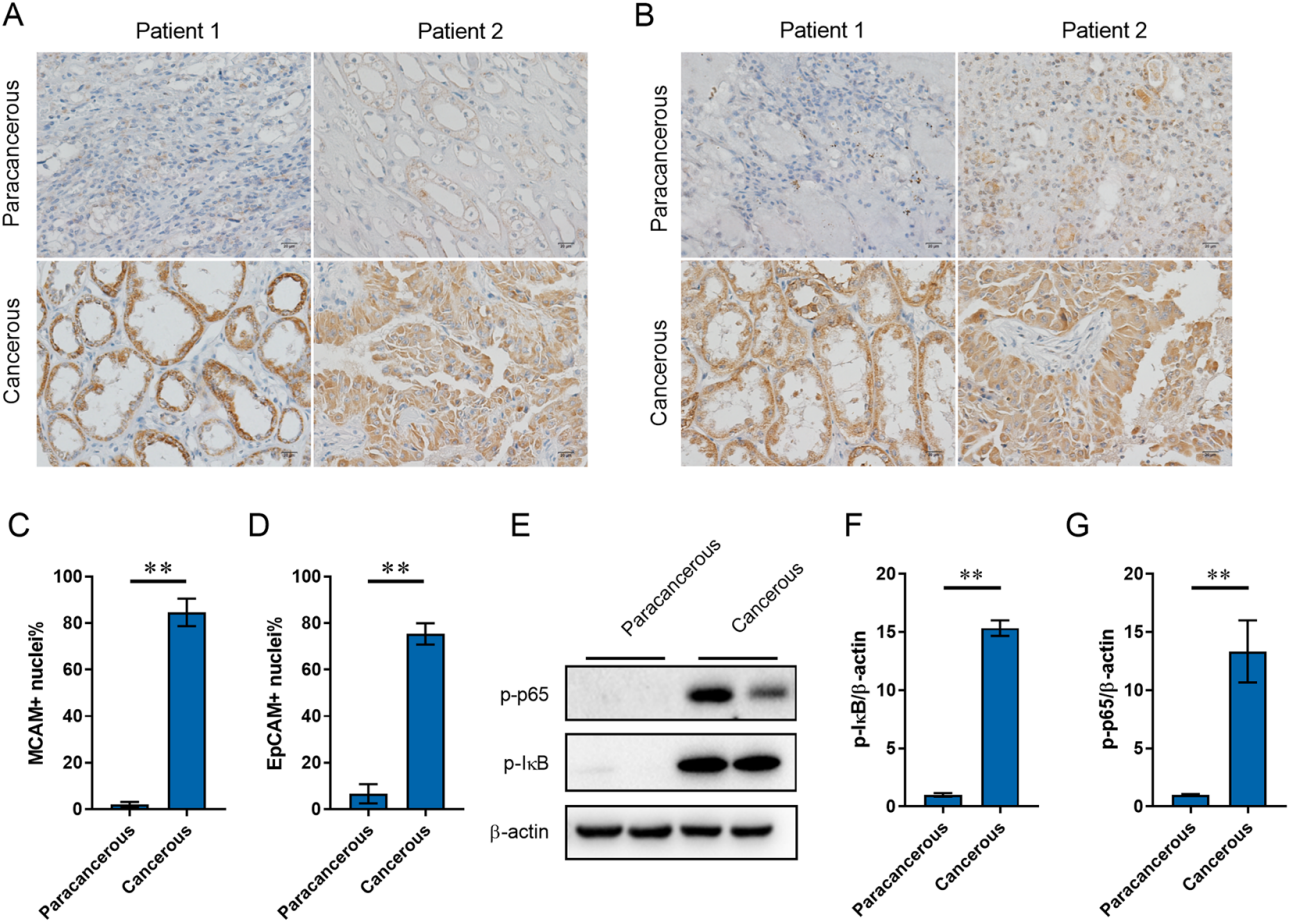
The distributions of adhesion molecules and activation of NF-κB in cancerous and paracancerous tissues. (**A**–**D**) The distribution of MCAM and EpCAM in cancerous and paracancerous tissues was determined using IHC. (**A**,**C**) MCAM; (**B**,**D**) EpCAM. (**E**–**G**) Phosphorylated p65 and p-I-κB in cancerous and paracancerous tissues was measured using Western blot. (**E**) A representative gel for p-p65 (upper panel), p-I-κB (middle panel) and β-actin (lower panel) was shown. (**F**) p-I-κB/β-actin. (**G**) p-p65/β-actin. The grouping of blots cropped from different gels of same samples. All data were expressed as means ± S.E.M. (N = 8). **P < 0.01.

Serum CRP was analyzed in RCC patients and controls. As shown in Fig. 2A, there was a rising trend on serum CRP level in RCC patients. Serum adhesion molecules were then analyzed in RCC patients and controls. Interestingly, serum ICAM was elevated in RCC patients (Fig. 2B), whereas there was no difference on serum VCAM levels between two groups (Fig. 2C). As shown in Fig. 2D, there was a rising trend on serum MCAM. Laminin alpha 4 (LAMA4) is extracellular matrix interaction partner of MCAM^22^. As shown in Fig. 2E, the levels of serum LAMH4 were higher in RCC patients than those in controls. In addition, serum EpCAM was higher in RCC patients than that of controls (Fig. 2F). The distribution of MCAM and EpCAM, two adhesion molecules in cancerous tissues, was then determined among RCC patients. As expected, the expression of MCAM were much higher in cancerous tissue than that of paracancerous tissue (Fig. 3A,C). In addition, the expression of EpCAM in cancerous tissues was significantly higher than that of paracancerous tissue (Fig. 3B,D).

### NF-κB is activated in cancerous tissues among RCC patients

Phosphorylated I-κB and NF-κB p65 were measured in cancerous tissue. As shown in Fig. 3E,F, the levels of p- IκB were higher in cancerous tissue than in paracancerous tissue. The levels of p-p65 were accordingly higher in cancerous tissue than in paracancerous tissue (Fig. 3E,G).

### Vitamin D status is reversely associated with serum CRP and adhesion molecules in RCC patients

**Figure 4:**
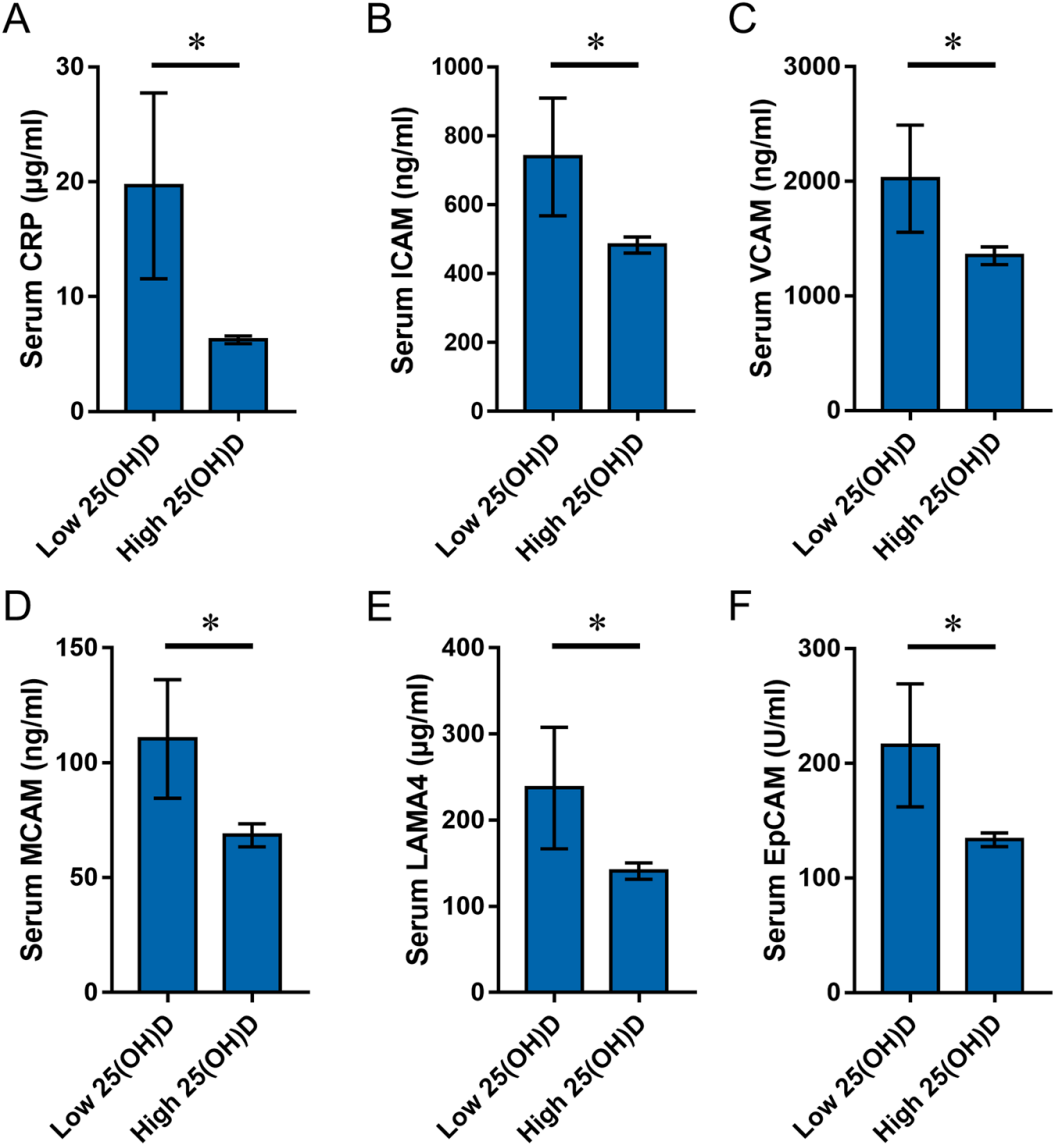
Low vitamin D status is associated with serum CRP and adhesion molecules in RCC patients. RCC patients were divided into two groups according to serum 25(OH)D level: >20 ng/ml or <20 ng/ml. The levels of serum CRP and adhesion molecules were compared between two groups. (**A**) CRP; (**B**) ICAM; (**C**) VCAM; (**D**) MCAM; (**E**) LAMA4; (**F**) EpCAM. All data were expressed as means ± S.E.M. *P < 0.05.

**Figure 5:**
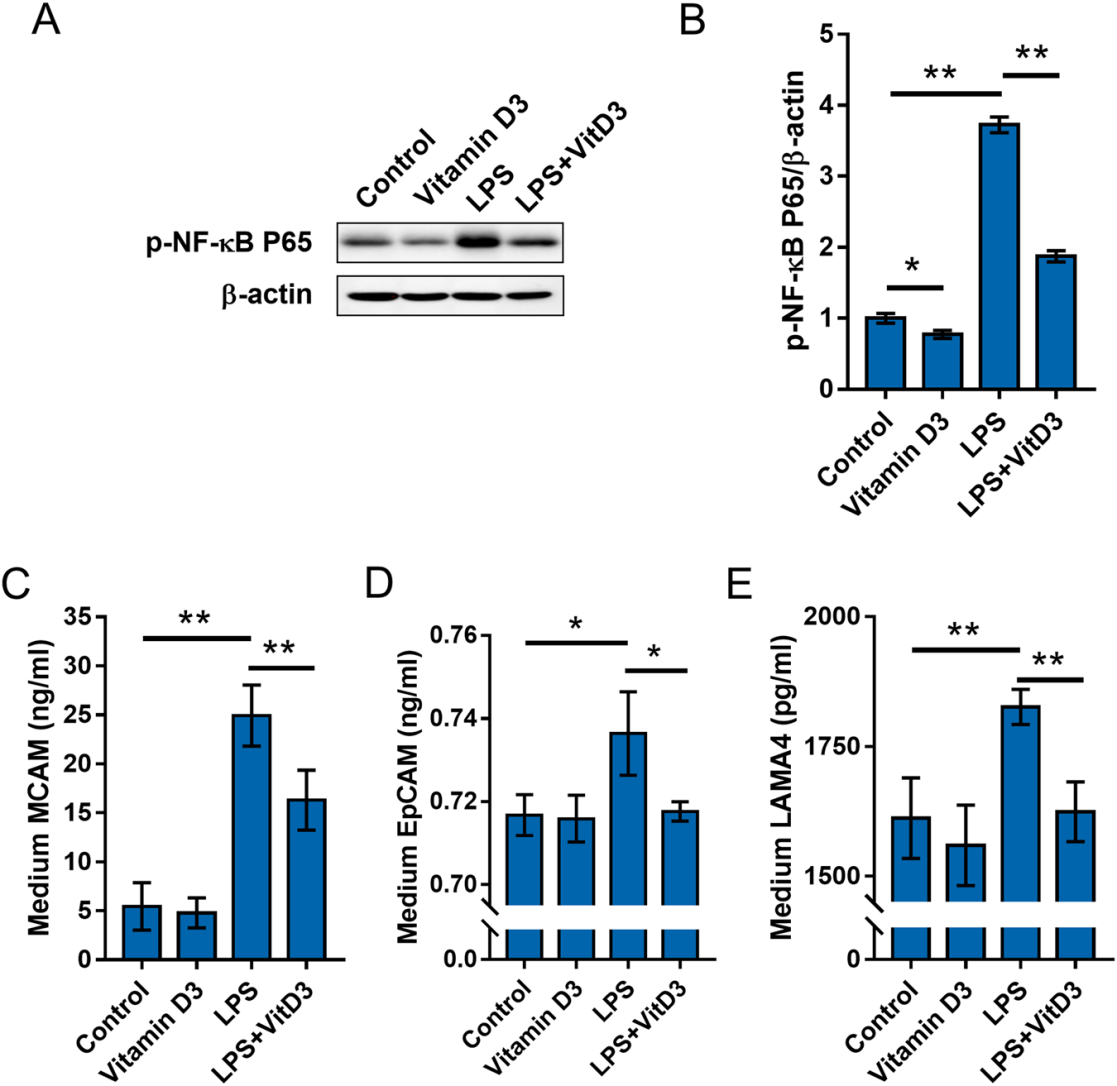
The effects of vitamin D3 on LPS-stimulated NF-κB and adhesion molecules activation in RCC cells. (**A**,**B**) ACHN cells were pretreated with 1,25(OH)2D3, the active form of vitamin D3, and then stimulated with LPS for 6 h. Phosphorylated p65 was measured using Western blot. (**A**) A representative gel for p-p65 (upper panel) and β-actin (lower panel) was shown. (**B**) p-p65/β-actin. All experiments were repeated for six times. The grouping of blots cropped from different gels of same samples. All data were expressed as means ± S.E.M. (N = 6). **P < 0.01. (**C**–**E**) ACHN cells were pretreated with 1,25(OH)2D3, the active form of vitamin D3, and then stimulated with LPS for 6 h. The level of MCAM, LAMA4 and EpCAM in medium was measured using ELISA. (**C**) MCAM in medium; (**D**) LAMA4 in medium; (**D**) EpCAM in medium. All data were expressed as means ± S.E.M. (N = 8). **P < 0.01.

Limited by the sample size, we defined patients with VDD (serum 25(OH)D < 20 ng/ml) as a low vitamin D status, while patients with 25(OH)D ≥ 20 ng/ml is defined as a relatively high status. All RCC patients were divided into two groups according to serum 25(OH)D level: H-VitD (≥20 ng/ml) or L-VitD (<20 ng/ml). Serum CRP level was compared between two groups. As shown in Fig. 4A, serum CRP level was significantly higher in RCC patients with L-VitD than those with H-VitD. The correlation between serum 25(OH)D and CRP levels was then analyzed among RCC patients and controls. As expected, serum 25(OH)D was not correlated with CRP among controls. Interestingly, serum 25(OH)D was reversely correlated with serum CRP among RCC patients (Table 4; *r* = −0.27, *P* < 0.05). Serum adhesion molecules were analyzed. As expected, the levels of all detected adhesion molecules were higher in RCC patients with L-VitD than those with H-VitD (Fig. 5B–F). The link between serum 25(OH)D and adhesion molecules was then analyzed. As expected, no link between serum 25(OH)D and all adhesion molecules was observed in controls (Table 4). Of interest, serum 25(OH)D was reversely correlated with serum ICAM (*r* = −0.25, *P* < 0.05), VCAM (*r* = −0.23, *P* < 0.05), MCAM (*r* = −0.27, *P* < 0.05), LAMA4 (*r* = −0.23, *P* < 0.05) and EpCAM (*r* = −0.25, *P* < 0.05) in RCC patients (Table 4).

**Table 4:** Association between serum 25(OH)D and adhesion molecules.

	CRP	sICAM	sVCAM	MCAM	LAMA4	EpCAM
Control	0.20	−0.11	0.05	0.19	0.04	0.02
RCC	−0.27^a^	−0.25^a^	−0.23^a^	−0.27^a^	−0.23^a^	−0.25^a^

^a^P < 0.05.

### Vitamin D3 inhibits NF-κB activation and MCAM upregulation in RCC cells

To further explore the mechanism through which low vitamin D status upregulates expression of adhesion molecules among RCC patients, ACHN cells were pretreated with 1,25(OH)2D3, the active form of vitamin D3, and then stimulated with LPS to observe whether active vitamin D3 inhibits adhesion molecules in RCC cells. As expected, the levels of MCAM, LAMA4 and EpCAM were elevated in LPS-stimulated ACHN cells (Fig. 5C–E). Interestingly, pretreatment with 1,25(OH)2D3 suppressed LPS-evoked elevation of MCAM, LAMA4 and EpCAM in ACHN cells (Fig. 5C–E). The effects of pretreatment with 1,25(OH)2D3 on LPS-induced NF-κB activation were measured. As expected, the level of phosphorylated NF-κB was significantly elevated in LPS-stimulated ACHN cells (Fig. 5A,B). Interestingly, pretreatment with 1,25(OH)2D3 inhibited LPS-induced activation of NF-κB in ACHN cells (Fig. 5A,B).

### Vitamin D3 promotess integration between VDR and NF-κB p65 subunit in RCC cells

**Figure 6:**
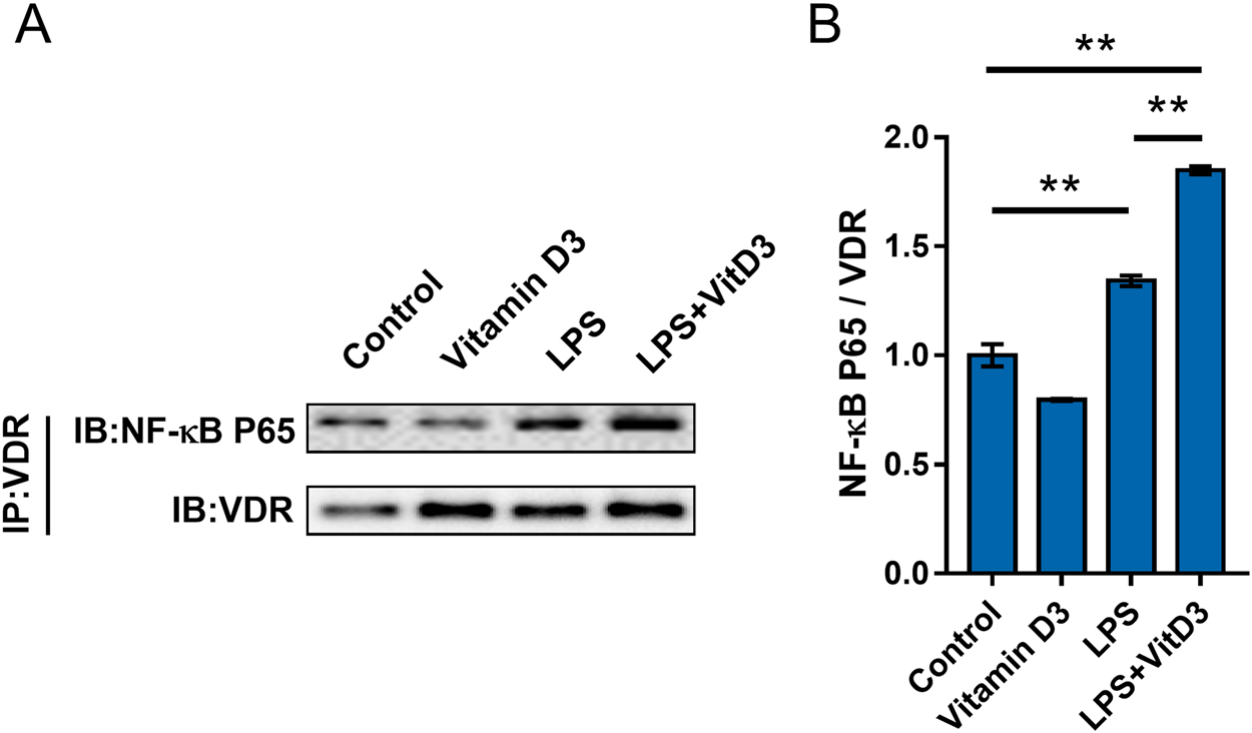
Vitamin D3 reinforces interaction between VDR and NF-κB p65 subunit in RCC cells. ACHN cells were pretreated with 1,25(OH)2D3, the active form of vitamin D3, and then stimulated with LPS for 6 h. The interaction between NF-κB p65 and VDR was detected using CoIP. All experiments were duplicated for four times. A representative gel for NF-κB p65 (upper panel) and VDR (lower panel) was shown. The grouping of blots cropped from different gels of same samples. All data were expressed as means ± S.E.M. (n = 4). **P < 0.01.

The integration between VDR and NF-κB p65 subunit in RCC cells was measured by CoIP. As shown in Fig. 6, 1,25(OH)2D3 plus LPS elevated level of p65 in immunocomplexes precipitated by anti-VDR antibody. These results suggest an integration between VDR and NF-κB p65 subunit in RCC cells.

## Discussion

Several epidemiological reports indicated that occupational ultraviolet exposure was negatively correlated with risk of RCC in different population^47,48^. A large prospective cohort study demonstrated that plasma 25(OH)D level was reversely associated with the incidence of RCC in men and women^37^. According to a report from the European Prospective Investigation into Cancer and Nutrition, circulating 25(OH)D was negatively associated with Odds Ratio of RCC after adjustment for confounding factors^49^. A recent study demonstrated that circulating 25(OH)D was positively associated with survival of kidney cancer^50^. The present study analyzed the link of low vitamin D status with RCC risk. We found that circulating 25(OH)D in RCC patients was lower than that of controls. Multivariable logistic regression analysis indicated an inverse link between RCC and serum 25(OH)D after adjustment for confounding factors. These results suggest that low vitamin D status is linked with an increased RCC risk.

Indeed, all RCC patients recruited in this study are new cases, most of which belong to T1a/b (34/50, 68%) and G1/2 (30/50, 60%). Thus, additional research is necessary to analyze the correlation between serum 25(OH)D level and RCC severity in a large sample of population. The methods for determining serum 25(OH)D levels include HPLC, LC-MS/MS, RIA and ELISA. The LC-MS/MS is the most accurate method that can simultaneously measure serum 25(OH)D2 and 25(OH)D3. LC-MS/MS is usually used a gold standard. However, LC-MS/MS method is time-consuming. In addition, specimen pretreatment for LC-MS/MS is complicated and the results are greatly influenced by human factors. On the other hand, the RIA method is characterized by high sensitivity, specificity and precision, and low requirements for equipment and equipment. In addition, the RIA method has good consistency with LC-MS/MS method and is often used to detect serum 25(OH)D in a large sample of population^51,52^. Thus, the RIA method is used to detect serum 25(OH)D in this study.

Numerous reports suggest that inflammation plays an important role in RCC metastases and progression^53,54^. CRP, a marker of inflammation, is an independent prognostic factor for overall survival of RCC patients^8,55^. An early report showed that an increase in preoperative CRP was linked with poor survival in patients with localized RCC^56^. According to a recent study, serum CRP level is a strong independent factor associated with survival of RCC patients treated with sunitinib^57^. This study analyzed the link between 25(OH)D and CRP in RCC patients. We found that serum CRP in RCC patients with L-VitD was higher than those with H-VitD. Further analysis found that serum CRP level was reversely correlated with serum 25(OH)D level among RCC patients. The study indicates that low vitamin D status is linked with increased serum CRP among RCC patients.

Accumulating data have demonstrated that adhesion molecules are prognostic molecular markers in RCC patients. Two early studies showed that patients with short survival or advanced RCC had higher levels of serum ICAM-1 than low-grade and/or low-stage RCC patients^58,59^. Another report indicated that VCAM-1, commonly overexpressed in RCC, was involved in tumor immune evasion^60^. According to a recent study, MCAM and its extracellular matrix interaction partner LAMA4, highly expressed in locally advanced tumors as well as secondary metastases, were predictive markers for poor RCC prognosis^22^. The present study showed that serum ICAM, LAMA4 and EpCAM were higher in RCC patients than in controls. Importantly, MCAM and EpCAM were highly expressed only in cancerous tissue but not in paracancerous tissue. Further observation found that the levels of all detected adhesion molecules were higher in RCC patients with L-VitD than those with L-VitD. These results suggest that low vitamin D status is associated with increased expression of adhesion molecules among RCC patients.

The mechanism through which VDD upregulates expression of adhesion molecules among RCC patients remains obscure. An *in vitro* study demonstrated that active VitD3 downregulated expression of ICAM-1 and VCAM-1 in endothelial cells through suppressing NF-κB activation^61^. According to an *in vivo* report, pretreatment with VitD3 attenuated LPS-induced upregulation of renal ICAM-1 and VCAM-1 by inhibiting activation of NF-κB in the kidneys^62^. In the present study, we found that nuclear VDR was low in cancerous tissue. By contrary, renal NF-κB was activated only in cancerous but not in paracancerous tissue. Correspondingly, renal adhesion molecules were highly expressed only in cancerous tissue but not in paracancerous tissue. The *in vitro* experiments found that VitD3 blocked activation of NF-κB and suppressed upregulation of adhesion molecules in RCC cells. Moreover, VitD3 suppressed NF-κB through promoting the integration between VDR and NF-κB p65 in RCC cells, which provides a mechanistic explanation for linking VDD, low VDR and increased expression of adhesion molecules in RCC patients.

The present study placed emphasis on analyzing the association of low vitamin D status with serum CRP and adhesion molecules among RCC patients. We demonstrated that there was a link among low vitamin D status, cancerous NF-κB activation and increased expression of adhesion molecules in RCC patients. However, the present study is observational. The findings herein described are not concretely able to demonstrate a causal association between both CRP and adhesion molecules and the prognosis of RCC patients with low vitamin D status. Several studies have demonstrated that serum calcium level is a factor of prognosis^62^. Thus, further studies are required to identify a causal association between both CRP and adhesion molecules and the prognosis of RCC patients with low vitamin D status. In addition, an *in vivo* experiment may be needed to measure the effects of supplementation with VitD3 on serum CRP and adhesion molecules as well as NF-κB signaling in RCC animal model.

In summary, the present study analyzed the association of low vitamin D status with serum CRP and adhesion molecules in RCC patients. We showed that serum 25(OH)D level was reduced among RCC patients. By contrast, the expression of renal adhesion molecules was upregulated among RCC patients. Moreover, vitamin D status was reversely correlated with serum CRP and adhesion molecules among RCC patients. We observed that NF-κB in cancerous tissue was activated among RCC patients. The *in vitro* experiments found that active VitD3 suppressed NF-κB activation and adhesion molecules through promoting integration between VDR and NF-κB p65 in RCC cells. Our results suggest a link among low vitamin D status, local inflammation and increased expression of adhesion molecules in RCC patients.
